# Factors Influencing Young People’s Intention toward Municipal Solid Waste Sorting

**DOI:** 10.3390/ijerph16101708

**Published:** 2019-05-15

**Authors:** Lin Shen, Hongyun Si, Lei Yu, Haolun Si

**Affiliations:** 1 Humanities and Management School, Hebei Agriculture University, Cangzhou 061100, China; shenlin@hebau.edu.cn (L.S.); wgxy@hebau.edu.cn (L.Y.); sihaoluns@163.com (H.L.S.); 2 School of Economics and Management, Tongji University, Shanghai 200092, China

**Keywords:** young people, intention, MSW, critical factor, theory of planned behavior

## Abstract

With the rapid growth of urban economy and population in China, the output of municipal solid waste (MSW) has dramatically increased becoming a constant threat to residents’ living environment and health. The classification intention of residents plays a pivotal role in solving the problem of MSW disposal. While numerous studies have examined the classification behavior of MSW from the perspective of ordinary residents and households, few studies have attempted to understand young people’s sorting intention. The novelty of this research is to explore the determinants that affect young people’s intention toward municipal solid waste sorting (MSWS) by extending the predictive factors of environmental concern and personal moral obligation into the theory of planned behavior (TPB). A sample of 524 young respondents from Hebei Province in China were used to conduct a structural equation model (SEM) validation. The empirical results revealed that, according to the rankings of significance, personal moral obligation, perceived behavioral control, and subjective norm had positive influences on young people’s intention toward MSWS, while attitude and environmental concern did not. Furthermore, the multi-group comparison showed that, compared with the male and rural group, the intention of female and urban respondents to classify MSW was not affected by subjective norms. Some targeted managerial implications were ultimately proposed to promote young people’s intention toward MSWS. This study contributes to the existing knowledge system of MSWS by revealing the classification intention of young people as a group. The findings and implications provide the government with useful insights for encouraging young people to actively participate in MSWS.

## 1. Introduction

In recent years, disposal of municipal solid waste (MSW) has attracted worldwide attention, especially in countries and regions with high population density, such as India, Japan, and China [1]. How to deal with MSW is a great challenge in both urban and rural areas of China [2]. In 2016, the total amount of MSW that was collected and transported reached 188.51 million tons in 214 cities in China [3]. The total amount of MSW in 2017 reached 300 million tons in rural China, which nearly exceeded the urban level [4].

Municipal solid waste sorting (MSWS) is particularly important for the human living environment and physical health because domestic waste contains a large amount of hazardous waste and corrosive substances, which indirectly threaten human health by polluting land, water or air. Developed countries have done a great deal in terms of MSWS [2]. The number of MSW separation categories in Japan reaches up to 25 [5], and the recycling rate of household waste in Germany is as high as 60% [6]. Many cities in China, such as Beijing, Hangzhou, and Chengdu, have successively launched MSWS activities to deal with the increasingly serious problem of MSW [7]. However, the current situation remains unsatisfactory. The recycling rate of MSW disposed by public sectors is less than 2% in 2015 [8]. Since human behavior is influenced by the interaction between organizations and the environment, examining the intention of people in a particular organization can improve the ability of managers to predict and direct individual behaviors. Therefore, to improve the recycling efficiency of household garbage, it is necessary to investigate the influencing factors of MSWS from the perspective of individual behavior and intention.

Numerous studies have investigated individual behavior and intention toward MSWS in relation to different countries and regions, cultures, income levels, and environmental conditions. These studies mainly aimed at groups of ordinary residents or households [9,10,11,12,13,14,15,16,17], but few have specifically tried to understand young people’s intention toward MSWS. As masters of the future society, well-educated young people are the most important group, and their behavioral intentions are crucial to the sustainability of the future living environment. In addition, developing the habit of MSWS is a long-term process, regardless of the relationship between age and garbage classification intention, and we should focus on cultivating the awareness of MSWS in the younger generation.

Although scholars such as Zhang et al. [2] and Wan et al. [18] conducted a survey to reveal the factors influencing students’ behavior of MSWS on university campuses in Beijing and Hong Kong, respectively, the scope of these studies is limited to college students. In fact, young people include mainly individuals between 15 and 30 years old [19,20], and this group has a broader range and a more decentralized education level than the college students group. Furthermore, young people include individuals of different genders, household registrations and income levels, and what is not yet clear is whether there are differences between different groups.

Therefore, the primary aims of this study are: (1) to implement a questionnaire survey on MSWS for the group of young people; (2) to explore the critical factors influencing young people’s intention toward MSWS; (3) to understand the potential impact differences between various groups, such as different genders and household registrations; and (4) to propose some targeted governance strategies for improving the classification willingness of young people. To achieve these objectives, we first develop research hypotheses based on previous studies. Considering that well-educated young people may be more concerned about the environment and more responsible at the personal moral level, we incorporate environmental concern and personal moral obligation into the theory of planned behavior (TPB) and propose a comprehensive theoretical model. Next, the structural equation model (SEM) method is employed to test out research hypotheses and conduct a multi-group comparison of valid data from 524 young respondents from Hebei Province, China. Lastly, empirical results are compared and discussed with those of previous studies to provide useful insights.

The remaining parts of the paper are organized as follows: Section 2 provides the literature review and hypothesis development. Section 3 illustrates the data collection and research method. Section 4 presents the results of model reliability and validity analysis, goodness-of-fit analysis, hypothesis testing, and multi-group comparison. Section 5 discusses the results and proposes some management strategies. Section 6 concludes with findings and the contributions of this study.

## 2. Literature Review and Hypothesis Development

Pro-environmental behavior refers to behaviors that can reduce ecological damage, protect natural resources, and improve environmental quality [21,22]. The TPB proposed by Ajzen in 1985 [23] has been widely adopted to investigate pro-environmental behavior [24]. This theory is derived from the theory of reasoned action (TRA), which provides an important analytical framework for understanding and predicting individual social behavior. In 1991, Ajzen published an article titled “Theory of Planned Behavior” [25], which marked the maturity of this theory. According to TPB, individual behavior is mainly determined by intention, which is mainly influenced by three factors: attitude (ATT), subjective norm (SN), and perceived behavioral control (PBC) [25]. That is, the more positive the ATT is, the stronger the SN and PBC will be; thus, the behavioral intention of an individual can strengthen [24].

Over the past few decades, TPB has been widely used in many fields, such as human social behavior, marketing and health-related behavior. A large number of meta-analytic reviews have confirmed that individual BI and actual behavior can be well explained and predicted by TPB [26,27,28,29]. TPB is also used to investigate individual pro-environmental behavior, such as pollution reduction intention [20,30], choice of travel mode [31,32], energy-saving behavior [33,34,35], domestic waste sorting, and recycling behavior [36,37,38,39,40]. While exploring the influencing factors of an individual’s intention and behavior, these studies also confirm the applicability of TPB in the field of pro-environmental behavior.

Although the efficacy and applicability of the TPB have been verified in previous studies, the TPB has been criticized for its lack of sufficient assumptions regarding individual intention and behavior. Therefore, other variables should be included to improve the interpreting ability of the TPB [36,41]. Ellen et al. [42] and Zsoka [43] proposed that individuals with a high degree of environmental concern have a strong willingness to protect the environment. Thus they expanded environmental concern based on basic TPB and confirmed the important influences of environmental concern on one person’s pro-environmental behavior. In addition, Gold [44] and Kaiser [45] purported that an individual’s willingness and behavior can be better predicted with a model that includes moral obligation, which should also be incorporated into TPB. Greaves et al. [46] and De Leeuw et al. [47] added descriptive norms into TPB to identify critical factors that influence the pro-environmental behavior of consumers. Zheng et al. [48] and Shi et al. [49] combined the technology acceptance model (TAM) with TPB to investigate the willingness of young people to rent a house and use urban-shared products, respectively. These additional variables expand and increase the efficiency of TPB in interpreting and predicting intention and behavior in specific situations.

**Figure 1:**
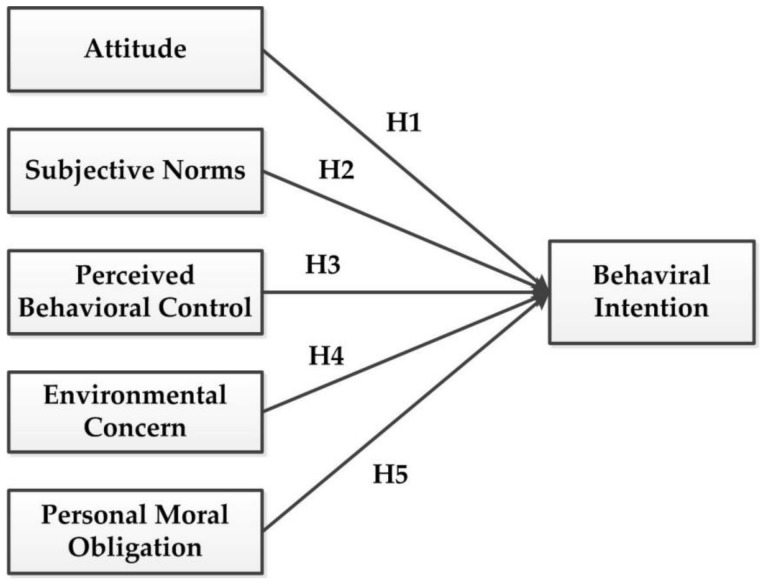
Extended theoretical model.

In accordance with the original TPB, this study adds two latent variables, namely, environmental concern (EC) and personal moral obligation (PMO), and constructs a model with behavioral intention (BI) as a dependent variable to explore the influencing factors of young people’s intention toward MSWS. Figure 1 displays the extended theoretical model.

### 2.1. Attitude

ATT refers to an individual’s positive or negative attitude in performing a particular behavior [29]. As ATT is a relatively persistent and stable psychological construct, many studies have confirmed the influence and prediction of ATT on BI [50,51]. Most studies have shown that individuals who are positive toward a certain behavior likely have a strong willingness to participate [52,53]. This study defines ATT as young people’s perceptions and tendencies of behavior toward MSWS. If young people hold a positive attitude toward MSWS, then they become more aware of the importance of MSWS and are consequently more intent on engaging in MSWS, and vice versa [54]. Therefore, we propose the following hypothesis:

**H1.**  ATT is positively related to MSWS intention.

### 2.2. Subjective Norm

SN refers to the effect of external social pressure on a subject’s specific behavior [29]. SN is divided into prescriptive and demonstrative [55]. The former is mainly derived from unit leaders, government workers, and other authorities. They have strong leadership and driving roles, which have an important impact on individual decision-making. The latter is derived from social resources, such as family members, neighbors, and friends, who provide important references and examples for an individual during decision-making. Previous studies have shown that the greater the external social pressure, the stronger the willingness of an individual to participate [29,56]. In the context of East Asian culture, society encourages collectivism rather than individualism [57]. Thus, individuals are easily influenced by leaders and even related organizations. In this study, SN refers to the influence of external social pressure on the willingness of young people to classify MSW. The greater the social pressure that young people perceive from the classification of MSW, the stronger their willingness to participate [35]. Thus, we propose the following hypothesis:

**H2.**  SN is positively related to MSWS intention.

### 2.3. Perceived Behavior Control

PBC refers to a person’s perception of the difficulty in performing a particular behavior [25]. According to Ajzen [24,58], PBC includes two aspects, namely, control belief and perceived intensity. The former refers to various factors that restrain or promote behavior, and the latter corresponds to individual self-efficacy. The control belief of young people in MSWS mainly comes from factors perceived to inhibit or promote their participation in garbage classification, and some of these factors include time, energy, and other resource constraints. Perceived intensity refers to the self-efficacy that young people perceive from MSWS, that is, the self-confidence of individuals in their ability to participate in MSWS [59]. The more confident young people are in their ability to classify MSW, the stronger their willingness to participate in MSWS. Based on the above arguments, we propose the following hypothesis:

**H3.**  PBC is positively related to MSWS intention.

### 2.4. Environmental Concern

EC is divided into two categories [38]. The first one involves specific environmental issues, such as attitudes toward water pollution. The second one focuses on comprehensive and universal environmental issues, such as attitude toward the relationship between humans and the environment. Previous studies have shown that people with high levels of EC are more willing to respond to environmental issues and apply pro-environmental behaviors [60,61]. Maichum et al. [62] confirmed that EC has a positive impact on consumers’ willingness to purchase green products. This study defines EC as a holistic and comprehensive view of environmental issues. If young people care about the environment and realize that their actions continuously affect the environment, then they are more willing to take pro-environmental actions. In view of the above analysis, we propose the following hypothesis:

**H4.**  EC is positively related to MSWS intention.

### 2.5. Perceived Moral Obligation

PMO refers to the subjective judgment of an individual on whether to act in a certain way or not [63]. PMO reflects individual self-expectation and attitude toward specific behaviors, which are derived from individual norms and values [64]. In contrast to SN, which is mainly derived from external social pressure, the impact of PMO on individual behavior mainly comes from internal pressure, such as responsibility and obligation. Individuals feel proud if their actions are consistent with PMO; otherwise, they feel guilty [65]. PMO can also significantly increase the proportion of interpretation variance in the original TPB model [31,35,51]. Ru et al. [20] and Wan et al. [18] added PMO to TPB to analyze young people’s intention to reduce PM2.5 and staff to classify takeaway waste. Their results show that PMO has a significant positive impact on pro-environmental BI. In this study, PMO refers to young people’s subjective judgment of MSWS, which includes sense of responsibility, obligation, and guilt if they do not classify MSW. In theory, if young people consider that they have the responsibility and obligation to classify MSW, then their intention strengthens. Thus, we propose the following hypothesis:

**H5.**  PMO is positively related to MSWS intention.

## 3. Methodology

### 3.1. City Background 

**Figure 2:**
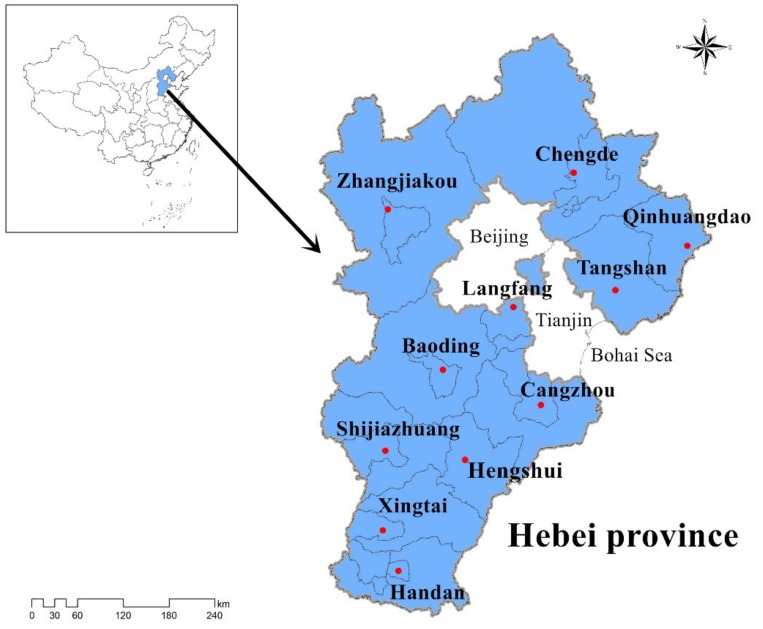
Location of Hebei, China.

The questionnaire surveys were conducted in Hebei Province, China. As shown in Figure 2, Hebei Province is bordered by Bohai Sea in the east and Beijing (the capital of China) in the inner ring. Its geographic location is longitude 113°27′–119°50′ and latitude 36°05′–42°40′. Hebei Province covers an area of 188,800 km^2^ with a resident population of 75.56 million (ranked 6th out of 31) at the end of 2018 [66].

According to statistics from the Hebei Provincial Bureau of Statistics, the total GDP of Hebei Province in 2018 was 536.38 billion USD (ranked 9th out of 31), and per capita disposable income was 3191.37 USD (ranked 17th out of 31). With the increase in population and the acceleration of urbanization, the waste in Hebei Province grows at an average annual rate of 10%. By the end of 2018, the annual amount of MSW in Hebei Province had reached over 11 million tons, with a daily average of over 30,000 tons.

To encourage MSWS and improve the living environment, The People’s Government of Hebei Province issued “Implementation opinions on further promoting the treatment of domestic garbage in the province” in 2011, and set Tangshan and Handan (two cities in Hebei Province) as pilot cities [67]. However, little difference has been made since the implementation of the policies. In 2019, Hebei Province issued “opinions on strengthening the classification of municipal solid waste” to promote the classification of MSW.

In summary, Hebei Province is a suitable sample for this study for the following reasons: First, the population and economy of Hebei Province are both upper middle class, which indicates that Hebei Province is representative of regions with a high population density and stable economic development. Second, problems such as a low participation rate and ineffective classification in Hebei Province as well as other regions should be solved to promote the classification of MSW. Third, previous studies on intention toward MSWS mainly focus on urban residents who live in first-tier cities such as Guangzhou and Hong Kong [18,38]. Little research employs a sample that includes rural residents. According to statistics from the National Bureau of Statistics, China’s rural population accounted for more than 40% of the population at the end of 2017 [68]. Therefore, a study on Hebei Province can be extended to other regions in China. Hence, this paper focuses on young people in Hebei Province to study the influencing factors of MSWS intention.

### 3.2. Questionnaires

This study employed questionnaires to collect relevant data. First, mature scales and questions were selected from relevant studies to construct observation variables and ensure the validity of the questionnaire. That is, the items included in the questionnaire were referenced and improved based on previous studies, which are listed in the last column of Table 1. Second, a focus group consisting of two professors, two graduate students, and two undergraduates performed a pre-test before the formal investigation. 26 young people were randomly selected to fill out the questionnaire and propose amendments and suggestions to ensure that the questions were easily understood and the results reliable and effective. Furthermore, there is reported evidence that a seven-point scale provides more diverse options, which in turn increases the probability of meeting people’s objective reality, and sample motivation revealed and described using a seven-point scale is more accurate [69,70]. Therefore, a seven-point Likert scale was used in our questionnaire survey, where one signifies “strongly disagree” and seven indicates “strongly agree”. Table 1 shows the survey items for data collection.

### 3.3. Data Collection

The data required for this study were collected from January 2019 to February 2019, and the questionnaire was completed by employing a combination of online and offline methods. Through Wechat and QQ, which are the most popular social networks in China, questionnaires were randomly distributed to young people aged 15 to 30 years. The snowball sampling method was also adopted in the online survey, and respondents were asked to send questionnaires to other respondents who met the overall characteristics of the samples. The offline method was obtained mainly through the direct questioning of respondents by 11 trained investigators from different regions. Yadav et al. [19] and Cheah et al. [82] revealed that the results of random sampling among young people and students aged 15 to 30 years are effective and reliable. A total of 220 questionnaires were distributed on the spot and 500 were distributed online. A total of 524 questionnaires, including 186 paper questionnaires and 338 online questionnaires, were obtained.

### 3.4. Research Method and Statistical Analysis

Structural equation modeling (SEM) was employed using AMOS 22.0 to verify the relationship between BI and ATT, SN, PBC, EC, PMO. SEM can integrate the factor and path analysis methods, effectively deal with the structural relationship among multiple variables, and overcome the collinearity among independent variables. Therefore, SEM is an effective method to verify the causal relationship between variables [49].

The detailed steps are as follows. Firstly, descriptive statistics were employed to inspect the socio-demographic characteristics of the interviewees. Next, reliability, convergent and discriminant validity were conducted using SPSS 18.0 to confirm the applicability of the proposed model. The indicators of the reliability test include composite reliability (CR) and Cronbach’s alpha (C.A.) [49]. The procedure of the validity test includes exploratory factor analysis (EFA) and confirmatory factor analysis (CFA). The standardized factor loading of every item was applied in EFA, and the average variance extracted (AVE) of every construct was calculated in CFA to verify the convergent validity of the model. At the same time, the correlation analysis was employed to examine the discriminant validity. Thirdly, the goodness-of-fit of the model was tested and the commonly recommended threshold of SEM included: χ^2^/df < 3 (the ratio of chi-square to the degree of freedom), GFI > 0.9 (goodness-of-fit index), AGFI > 0.9 (adjusted goodness-of-fit index), RMSEA < 0.08 (root mean square error of approximation), RMR < 0.08 (root mean square residual), NFI > 0.9 (normed fit index), RFI > 0.9 (relative index of fit), and CFI > 0.9 (comparative fit index). Fourthly, the relationships linking BI and the five constructs were validated and the hypotheses testing results were reported. Furthermore, a nonparametric bootstrap procedure including 10,000 bootstrap samples and a 95% confidence interval (CI) was conducted to verify the significance level [83]. Lastly, a multiple group comparison was conducted to evaluate the applicability of the proposed model among different groups.

## 4. Data Analysis and Results

### 4.1. Descriptive Characteristics

Among the participants, 42.94% (*n* = 225) were males and 57.06% (*n* = 299) were females. Most participants were urban residents (66.98%, *n* = 351), followed by rural residents (33.02%, *n* = 173). Most respondents were between 20 and 25 years old (57.44%, *n* = 301), followed by 26 to 30 years (30.15%, *n* = 158). 67.37% (*n* = 353) of the interviewees reported having a college or Bachelor’s degree as their highest educational level, followed by Master’s degree or Ph.D. (19.47%, *n* = 102). Most of the respondents were students (49.81%) and office workers (36.07%). Table 2 provides a detailed sample description.

### 4.2. Measurement Model: Reliability and Validity

EFA and CFA were conducted to verify the convergent validity of the data. According to the initial EFA findings, three items (one from EC (EC5), one from PMO (PMO4), and one from BI (BI6)) were deleted because their factor loadings were smaller than 0.50. After deleting these items, the constructs’ factor loadings were scaled from 0.501 to 0.979, which were higher than the recommended threshold of 0.50 [84]. Next, the retaining items were examined by CFA. The average variance extracted (AVE) ranged from 0.556 to 0.939, which is higher than the suggested benchmark score of 0.50 [84]. The results indicated that the convergent validity of the measurement items was strong.

Composite reliability (CR) and Cronbach’s alpha (C.A.) were employed to test the internal consistency of the items in each construct [49]. Table 3 shows that CA ranged from 0.826 to 0.968, and the CR varied from 0.829 to 0.987. Generally, CA and CR scores are no less than 0.70 [84,85]. Thus, the constructs’ reliability was verified.

Next, discriminant validity was employed to measure the degree of difference between the two different constructs (Table 4). The results showed that all of the factor correlations were lower than 0.8 and lower than the square roots of AVEs, which ensures the discriminant validity of the model [86]. In general, the proposed model showed the necessary validity and reliability and was ready for further analysis.

### 4.3. Structural Model: Goodness-of-Fit Statistics

The structural model was constructed using AMOS 22.0. First, model fitting resulting from structural analysis did not meet the recommended threshold, suggesting the re-specification of the proposed framework. Through many experiments on SEM, Charles found that most models can hardly meet the fitting criteria the first time because of the data deviation or problems in the model itself [87]. After the modification indices were applied, CFA was conducted again on the modified theoretical framework. Subsequently, the value expressed a better model fit (χ^2^ = 452.438, χ^2^/df = 2.029, GFI = 0.930, AGFI = 0.906, RFI = 0.957, NFI = 0.965, CFI = 0.982, RMSEA = 0.044, RMR = 0.079). All goodness-of-fit statistics were within the commended limitation, demonstrating a good model fit of the proposed model to the data. Table 5 shows the goodness-of-fit indices.

### 4.4. Hypotheses Testing

**Figure 3:**
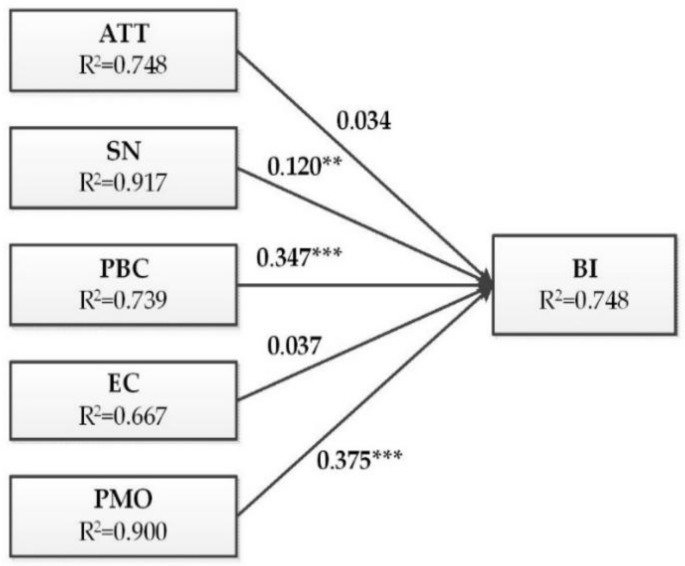
Path coefficients and significance of the research model.

Figure 3 shows that the comprehensive effect R^2^ value of BI was 0.748, which means 74.8% of the variance in BI can be interpreted by the model. The R^2^ values of ATT, SN, PBC, EC and PMO reached 0.917, 0.739, 0.667, 0.900 and 0.748, respectively, thus accounting for 91.7%, 73.9%, 66.7%, 90.0% and 85.5% of the variance in the homologous constructs. As a result, each construct can be well explained by the proposed model.

The results of the hypothesis test are shown in Figure 3 and detailed in Table 6. As shown in Table 6, the standardized path coefficients of SN, PBC and PMO were within the two-tailed 95% confidence interval [88], indicating that SN (β = 0.120, t = 3.113, *p* = 0.002), PBC (β = 0.347, t = 7.247, p < 0.001) and PMO (β = 0.375, t = 8.644, *p* < 0.001) have positive and significant effects on the separation intention of MSW. Thus, H2, H3 and H5 were supported. However, ATT (β = 0.034, t = 0.951, *p* = 0.342) and EC (β = 0.037, t = 0.876, *p* = 0.381) did not significantly affect intention. Hence, H1 and H4 were rejected.

### 4.5. Multiple Group Comparison

Multi-group comparison aims to evaluate whether the model that was adopted to a certain sample is suitable for other samples, that is, whether the proposed hypothesis model is applicable between different samples [85]. Multi-group comparison in this study involves gender and registration as moderator variables. The sample was divided into male and female groups on the basis of gender, and into urban and rural groups on the basis of registration. Four groups of samples were adopted to explore the applicability of the hypothesis model. The results of multi-group structural equation analysis and the confidence intervals are shown in Table 7.

With regard to the male and rural groups, the results show that SN, PBC and PMO have significantly positive effects on the intention toward MSWS, whereas ATT and EC do not. Furthermore, PBC and PMO had the greatest effects on classification intention. The results of the male and rural groups are consistent with those of all of the samples. However, as for the female and urban groups, although PBC and PMO have significant and positive impacts on the intention toward MSWS, SN, ATT and EC do not. Therefore, the results of the multi-group comparison reveal that SN has no significant effect on the classification intention of female and urban groups.

## 5. Discussion and Implications

### 5.1. PBC and Its Implications

PBC was proven to have significant and positive impacts on young people’s intention toward MSWS. Furthermore, according to path coefficient rankings, PBC contributes the second greatest effect among the five predictive factors. This result indicates that the degree of difficulty in MSWS is an extremely important factor for young people’s sorting intention. However, this finding is contrary to the conclusion of Ma et al. [59], who investigated public intention and behavior toward solid waste classification in under-developed areas of China. The reason may be that the college students and office workers surveyed in this study are relatively well educated, t are more confident in their participation in garbage classification, show a stronger sense of self-efficacy, and have a stronger willingness to classify garbage than senior individuals. Although we found the critical role of PBC on a person’s intention of MSWS, a comparative study by Ramayah [89] reported that PBC is not a significant influencing factor for recycling behavior. Therefore, while promoting the willingness of young people to recycle and classify, we should also consider transforming that willingness into actual behavior as much as possible.

Given the above findings, several measures should be implemented to enable young people to feel that the classification of MSW is easy to achieve. First of all, lectures can be held in classes, communities, and companies to popularize garbage classification standards and skills, thereby minimizing the difficulties involved in classifying garbage. Secondly, the supply of infrastructure for MSWS should be strengthened to facilitate the classification by young people. The relevant authorities should consider the characteristics of residential layout, population size, and other factors and arrange comprehensive and convenient MSWS facilities. Domestic garbage disposal facilities should be simple, easily identified, and reasonably located. Lastly, rewards should be given in accordance with the classification of MSW to enhance the willingness of young people to classify MSW.

### 5.2. SN, PMO, and Their Implications

SN and PMO significantly and positively affect young people’s intention toward MSWS. The effect of PMO is even greater at 0.375, which shows that the subjective judgment of the correctness of MSWS is the main factor that determines the willingness of young people to classify garbage. The conclusion of Ru et al. [20] about young people’s intention to reduce PM2.5 also confirms the important influence of PMO. When young people have a strong sense of responsibility and obligation toward MSWS, their garbage classification is strong.

Although SN significantly influences young people’s intention, the impact of utility is 0.120, which is lower than the impact of PMO. This result shows that external social pressure affects young people’s intention, but the impact is lower than the internal pressure felt by young people. However, a comparative study in Hong Kong by Wan et al. [18] is inconsistent with our finding. Wan et al. [18] claimed that SN has a stronger impact on intention toward MSWS than moral norms. The possible reasons for the contrary findings are as follows. The respondents in this study are basically post-90s. In comparison with post-80s and post-70s, they are more active in thinking, more independent in life, and have a stronger awareness of their independence. Therefore, they are not susceptible to external factors when making decisions. Second, young people in mainland China feel less external pressure because of the relatively low penetration rate of MSWS compared with that experienced by college students in Hong Kong. It was confirmed in Ho’s study [90] that social pressure is an important factor affecting recycling intention. If family members, friends, and colleagues classify MSW, then the herd mentality drives young people to be more involved in garbage classification.

The insight from the above findings is that full attention should be given to the cultivation of norms, values, and public awareness. Young people should realize that MSWS is a social behavior in which every citizen should actively participate. The classification of MSW can be included in the content of basic education planning so that MSWS can be integrated into teaching materials and classes and become one of the students’ practical contents. After receiving a relevant education, young people can grow up to consciously and forcefully promote the work of MSWS. In this manner, social civilization can be continuously promoted to create a cycle.

### 5.3. ATT, EC, and Their Implications

ATT has no significant positive impact on young people’s intention toward MSWS. This finding indicates that although young people recognize the importance of garbage classification, their intention has not significantly improved. In other words, young people think that classifying MSW is meaningful, but they may be unwilling to do it. The possible reasons are as follows. On the one hand, according to the respondents, since classification standards include classification between dry and wet, between organic and inorganic, and between recyclable and non-recyclable, these standards make respondents confused and unaware of how to classify MSW. Without professional guidance, young people are often unable to classify garbage. On the other hand, although laws and regulations concerning MSWS have been repeatedly issued, implementation remains vague due to unclear legal responsibilities. As mentioned in Loan et al. [91], after young people sort MSW and discharge it into designated garbage cans, the waste collectors mix the sorted waste with unsorted waste and uniformly dump them into a recycling car for transportation. As a result, young people’s initiative to classify MSW has been compromised, and they feel that they are doing useless work and are thus reluctant to classify MSW.

EC has no significant positive impact on young people’s intention toward MSWS, indicating that although young people are concerned about the environment, this concern does not contribute very much to their intention toward MSWS. The study by Wei et al. [92] on pro-environmental behavior supports our findings. That is, the public’s environmental knowledge and awareness of environmental protection is gradually increasing in China, but environmental protection behavior, especially active participation, is decreasing. The main possible reasons for this interesting conclusion are as follows. First, given the severity of the global environmental situation, the level of concern for environmental issues from the state to the individual continues to increase. However, from the perspective of young people, MSWS does not seem to bring about significant environmental improvement. Thus, they are more inclined to perform other effective pro-environmental behaviors, such as using green travel tools or buying green products [57,62]. Second, the problem of randomly discarding garbage is widely criticized in China because of education level, living habits, and for other reasons. Young people who are relatively well educated are willing to obey the rules and throw rubbish into bins. In fact, some young people believe that they have achieved environmental protection when they have put garbage in the trashcan, and whether they have classified it or not is unimportant.

According to normative focus theory, establishing good social norms is an important way of solving the problem of deviation between ATT, EC, environmental protection intention, and environmental protection behavior [93,94,95]. Therefore, the promotion of social norms should be fully exerted to enhance young people’s willingness to classify garbage. Television, newspapers, the Internet and social media should be employed to increase publicity and education about MSWS and to build a social atmosphere of “universal classification”.

In addition, the government should improve laws and regulations on MSWS, strengthen policy implementation, and form a long-term domestic garbage classification mechanism. Since March 2017, China has issued a number of national policies about MSWS, and various domestic management methods and implementation plans have also been introduced. First, domestic policies at all levels should be integrated on the basis of these methods and plans. Relevant laws and regulations should be systematized and completed. Second, policy implementation should be strengthened to implement a system of pollution responsibility. Individuals or organizations that refuse to classify MSW should be severely punished. Lastly, MSWS should be strictly implemented in the centralized garbage recovery process to avoid upstream secondary pollution.

### 5.4. Gender and Registration Differences and Their Implications

According to multi-group comparison, in addition to ATT and EC, the influence of SN on the intention of female and urban respondents is also not significant. In other words, external social pressure slightly affects the willingness of female and urban respondents to classify MSW compared with the male or rural group. Are women less susceptible to external pressure? A possible reason may be that women are becoming more independent and assertive as their education levels and labor participation rates increase. In teams, women are more likely to form small groups and generate opinions independently.

Compared with urban residents, rural residents have formed certain potential norms in the long-term communication process, which restrains their individual behaviors. The characteristics of the potential norm are based on informal norms and ethics. If an individual violates these norms, then the organization punishes the individual. This external pressure restrains the behavior of villagers, thereby ensuring the normal operation of the organization and improving organizational efficiency. However, as urban residents have relatively independent living spaces, they tend to be more independent in behavior and decision-making and are not easily affected by external pressure.

These findings indicate that different strategies could be applied to diverse groups. First, for young women, popularizing classification standards and increasing infrastructure supply can be conducted to reduce the difficulty in MSWS. At the same time, publicity and education can be adopted to enhance their sense of responsibility and thus improve their willingness to classify. Second, as for young men and rural young people, on the one hand, the restraining role of informal norms should be fully exerted. On the other hand, the legal system should be improved to replace the soft binding force of morality and reputation with the authority and mandatory binding force of laws and regulations. Lastly, for urban young people, the role of environmental protection organizations should be employed to promote young people’s intention toward MSWS.

### 5.5. Limitations

It is undeniable that there are several limitations in this study. First, the extended TPB model has only considered the context of EC and PMO. The present investigation is not specifically designed to evaluate factors that are related to habit, knowledge, and past experience. Second, with regard to the transformation of young people’s intention to behavior, subsequent verification is required on the basis of the observed behavioral results. Lastly, this study was carried out in the context of densely populated Chinese cities, while the situation in other countries and regions is not yet clear. These problems should be explored in future work.

## 6. Conclusions

Understanding young people’s motivation, intention, and influencing factors toward MSWS is of great theoretical and practical significance to guide them in actively participating in the classification of MSW. In this paper, we investigated the critical factors that influence young people’s intention toward MSWS by extending the TPB. The SEM method was employed to validate the research hypotheses of the proposed model, and a multi-group comparison was conducted to uncover the group differences in terms of different genders and different household registrations. Overall, this study made some interesting findings.

First, the results reveal that PMO and PBC are the most critical factors influencing young people’s intention toward MSWS, followed by SN. Second, contrary to our expectations, ATT and EC do not significantly contribute to young people’s intention toward MSWS. Third, compared with male and rural groups, female and urban groups’ intentions are not significantly affected by SN. On the basis of a comprehensive discussion, some targeted managerial implications were proposed to promote young people’s intention toward MSWS. For example, education and promotion programs should be commended to strengthen the awareness of classification, while laws and regulations should be improved and punishment of non-execution should be enforced. In addition, the supply of infrastructure should be strengthened and rationalized, and the classification of MSW should be included in the content of basic education planning to facilitate the formation of a long-term mechanism. Last, different strategies should be applied to diverse groups.

This paper represents one of the first attempts to thoroughly examine young people’s intention toward MSWS in China, and its findings contribute in several ways to our understanding of MSWS and could provide a basis for further research. Specifically, the testing results of the SEM confirm that the extended model has strong explanatory ability for investigating young people’s intention toward MSWS, which expands the application of TPB. More importantly, this paper describes the critical factors affecting young people’s intention toward MSWS and the differential impacts of different genders and different household registrations on classification intention; thus, our findings and implications could offer important insights for the government. In addition, since Hebei is a typical province in China in terms of population and economy, our research results and findings can be applied to guide young people’ garbage sorting practice in other areas of China, as well as other countries and regions with similar characteristics.

## Figures and Tables

**Table 1:** Survey items regarding young people’s intention toward MSWS.

Constructs	Measuring Items		Sources
**ATT**	ATT1	MSWS is a good idea.	[24,34,47]
	ATT2	MSWS is beneficial.	
	ATT3	MSWS is wise.	
	ATT4	MSWS is meaningful.	
SN	SN1	Most people who are important to me support MSWS.	[39,71,72]
	SN2	My family thinks that I should do MSWS.	
	SN3	My friends think that I should do MSWS.	
	SN4	My families and friends are doing MSWS.	
PBC	PBC1	MSWS is effortless.	[73,74,75]
	PBC2	Whether to classify MSW is entirely up to me.	
	PBC3	I have the relevant resources, time, and opportunity to perform MSWS behaviors.	
	PBC4	I can do MSWS as long as I want.	
EC	EC1	I think environmental issues are related to human survival.	[19,38,62,76]
	EC2	I think everyone should protect the environment.	
	EC3	Human activities affect the environment all the time.	
	EC4	Human beings must live in harmony with nature to survive.	
	EC5	I am concerned about the state of the environment.	
PMO	PMO1	Conducting MSWS behaviors is in line with my principles of environmental protection.	[49,77,78]
	PMO2	I believe that I have a moral obligation to conduct MSWS behaviors in daily life.	
	PMO3	I believe that I have a responsibility to conduct MSWS behaviors in daily life.	
	PNO4	I would feel guilty if I were not involved in MSWS behaviors in daily life.	
BI	BI1	I plan to take part in MSWS behaviors shortly.	[57,79,80,81]
	BI2	I will make an effort to take part in MSWS behaviors shortly.	
	BI3	I intend to isolate food waste separately when discarding.	
	BI4	I intend to isolate recyclable waste separately when discarding.	
	BI5	I intend to isolate dangerous waste separately when discarding.	
	BI6	I am willing to participate in MSWS behaviors shortly.	

**Table 2:** Demographic characteristics of the sample.

Feature	Type	Frequency	Percentage/%
Gender	Male	225	42.94
	Female	299	57.06
Register	Urban	351	66.98
	Rural	173	33.02
Age	15–19	65	12.41
	20–25	301	57.44
	26–30	158	30.15
Education	Senior high school or below	69	13.16
	College or Bachelor’s degree	353	67.37
	Master’s degree or Ph.D.	102	19.47
Profession	Student	261	49.81
	Office worker	189	36.07
	Other	74	14.12

**Table 3:** Reliability and convergence validity test results.

Constructs	Measuring Items	Mean	S.D.	Factor loading	C.A.	CR	AVE
ATT	ATT1	6.44	1.112	0.848	0.968	0.969	0.888
	ATT2	6.53	0.960	0.971			
	ATT3	6.51	1.021	0.979			
	ATT4	6.51	1.004	0.966			
SN	SN1	4.99	1.308	0.602	0.882	0.886	0.665
	SN2	4.60	1.555	0.896			
	SN3	4.69	1.526	0.907			
	SN4	4.16	1.579	0.821			
PBC	PBC1	5.11	1.414	0.856	0.826	0.829	0.556
	PBC2	4.94	1.594	0.501			
	PBC3	4.81	1.479	0.826			
	PBC4	5.48	1.376	0.747			
EC	EC1	6.15	1.115	0.879	0.962	0.964	0.869
	EC2	6.35	1.033	0.952			
	EC3	6.33	1.067	0.964			
	EC4	6.40	1.014	0.932			
PMO	PMO1	5.50	1.295	0.926	0.910	0.864	0.680
	PMO2	5.77	1.144	0.963			
	PMO3	5.77	1.158	0.966			
BI	BI1	4.81	1.567	0.775	0.914	0.987	0.939
	BI2	5.56	1.276	0.974			
	BI3	5.08	1.480	0.813			
	BI4	5.26	1.450	0.914			
	BI5	5.53	1.417	0.843			

**Table 4:** Correlation coefficient matrix and square roots of AVEs.

Constructs	ATT	SN	PBC	EC	PMO	BI
ATT	**0.942**					
SN	0.208	**0.815**				
PBC	0.333	0.518	**0.746**			
EC	0.566	0.282	0.444	**0.932**		
PMO	0.410	0.445	0.515	0.627	**0.825**	
BI	0.334	0.526	0.601	0.451	0.654	**0.969**

Note: Diagonal texts in boldface represent the square roots of AVE values.

**Table 5:** Measurement of model fit indices.

Fit Indices	Criteria	Before Model Correction	Model Adaptation Judgment	After Model Revision	Model Adaptation Judgment
χ^2^/df	<3	6.056	No	2.029	Yes
GFI	>0.9	0.781	No	0.930	Yes
AGFI	>0.9	0.734	No	0.906	Yes
RFI	>0.9	0.872	No	0.957	Yes
NFI	>0.9	0.885	No	0.965	Yes
CFI	>0.9	0.902	Yes	0.982	Yes
RMSEA	<0.08	0.098	No	0.044	Yes
RMR	<0.08	0.474	No	0.079	Yes

**Table 6:** Hypotheses results.

Hypothesis	Path Correlation	Standardized Path Coefficient	*t*-Value	Lo95	Hi95	*p*	Results
H1	ATT → BI	0.034	0.951	−0.039	0.102	0.342	NS
H2	SN → BI	0.120	3.113	0.041	0.196	0.002 **	Supported
H3	PBC → BI	0.347	7.247	0.257	0.434	***	Supported
H4	EC → BI	0.037	0.876	−0.054	0.123	0.381	NS
H5	PMO → BI	0.375	8.644	0.292	0.454	***	Supported

Note: * means *p* < 0.05, ** means *p* < 0.01, and *** means *p* < 0.001.

**Table 7:** Multi-group structural equation analysis results (standardized path coefficients).

Hypothesis	Path Correlation	Gender		Registration	
		Male CI (Lo95, Hi95)	Female CI (Lo95, Hi95)	Urban CI (Lo95, Hi95)	Rural CI (Lo95, Hi95)
H1	ATT → BI	0.077 (−0.126, 0.316)	0.016 (−0.056, 0.096)	−0.028 (−0.242, 0.151)	0.056 (−0.022, 0.135)
H2	SN → BI	0.192 ** (0.014, 0.385)	0.076 (−0.041, 0.194)	0.102 (−0.063, 0.264)	0.133 ** (0.009, 0.253)
H3	PBC → BI	0.436 *** (0.197, 0.985)	0.326 *** (0.192, 0.458)	0.395 *** (0.216, 0.723)	0.325 *** (0.188, 0.466)
H4	EC → BI	−0.121 (−0.396, 0.094)	0.091 (−0.012, 0.197)	0.077 (−0.111, 0.346)	0.023 (−0.077, 0.126)
H5	PMO → BI	0.402 *** (0.211, 0.845)	0.366 *** (0.242, 0.499)	0.358 *** (0.160, 0.584)	0.378 *** (0.242, 0.511)

## Abbreviations

AGFI	adjusted goodness-of-fit index
ATT	attitude
AVE	average variance extracted
BI	behavioral intention
C.A.	Cronbach’s alpha
CFA	confirmatory factor analysis
CFI	comparative fit index
CI	confidence interval
CR	composite reliability
EC	environmental concern
EFA	exploratory factor analysis
GFI	goodness-of-fit index
Lo95/Hi95	95% bias-corrected confidence intervals
MSW	municipal solid waste
MSWS	municipal solid waste sorting
NFI	normed fit index
PBC	perceived behavior control
PMO	personal moral obligation
RFI	relative fit index
RMR	root mean square residual
RMSEA	root mean square error of approximation
SEM	structural equation model
SN	subjective norm
TPB	theory of planned behavior
